# Draft Genome Sequence of the Murine Bacterial Isolate *Lactobacillus murinus* EF-1

**DOI:** 10.1128/genomeA.00077-17

**Published:** 2017-03-23

**Authors:** Eric Fritz, Michael J. Miller

**Affiliations:** Department of Food Science and Human Nutrition, University of Illinois at Urbana-Champaign, Urbana, Illinois, USA

## Abstract

Screening for lysogenic lactobacilli in rat fecal samples has identified *Lactobacillus murinus* EF-1. Whole-genome sequencing revealed a 2.30-Mb draft genome with 39.6% G+C content and 2,196 open reading frames. PHAST analysis identified three intact prophages of 26.1 kb, 25.4 kb, and 49.6 kb in size.

Lysogens, bacterial hosts with an embedded bacteriophage genome within their bacterial genome, account for approximately 40% of the total bacterial population across several microbial communities (1, 2). Identifying and characterizing lysogens with their intact viral genetic component enables further research to explore the roles that viruses play in aiding bacterial hosts in establishing and developing niches within gastrointestinal tract (GIT) environments (3). *Lactobacillus murinus* EF1 was isolated from a rat fecal sample and confirmed to be a lysogen by mitomycin C induction. *L. murinus* strains have previously been isolated and identified from rat, mice, porcine, canine, and humans (4, 5). Also, various *L. murinus* strains have been further characterized as probiotics in food formulations (4, 6, 7). With increased public interest in probiotics (7), the impacts of lysogenic bacteriophage on probiotic functions within the host GIT are beginning to unravel (2).

To identify punitive prophages found within *L. murinus* EF-1, genomic DNA was extracted. Mate-pair and paired-end libraries were then generated for Illumina MiSeq sequencing, resulting in 13,345,922 mate-pair and 9,992,616 paired-end sequencing reads. CLC Genomics Workbench *de novo* assembly version 8.5 (CLC Bio, Aarhus, Denmark) produced 18 scaffolds with a total length of 2,308,018 bp, a G+C content of 39.6%, and an *N*_50_ length of 309,081 bp; 99.46% of the sequenced mapped reads were assembled with 250× average coverage. RecA and HSP60 genes were used to confirm *L. murinus* identity (8, 9). Contigs were further analyzed and characterized using Prodigal (10) and RNAmmer rRNA (11) to confirm *L. murinus* open reading frames (ORFs) and rRNAs, respectively, and PHAST prophage (12) and CRISPRfinder (13) repeat identifier software to locate potential prophage genes and genomes. The initial annotation of *L. murinus* revealed 2,196 ORFs and eight subunits of rRNA. Three intact prophages were identified through PHAST, two on scaffold 6 and one on scaffold 7, along with four incomplete prophages on scaffolds 1 (6.9 kb), 2 (8.5 kb), 3 (19.7 kb), and 5 (9.8 kb). Also, one questionable CRISPR spacer was identified in scaffold 3. The identification of three intact prophages, four incomplete prophages, and a questionable CRISPR array may demonstrate a prophage preference of this strain, suggesting an environmental prophage advantage in the rat GIT for this strain. Future studies will characterize the lysogenic phages within *L. murinus* EF-1.

## Accession number(s).

This whole-genome shotgun project was deposited in GenBank under the accession number MPSN00000000. The version described in this paper is the first version, MPSN01000000.
